# SeADL: Self-Adaptive Deep Learning for Real-Time Marine Visibility Forecasting Using Multi-Source Sensor Data

**DOI:** 10.3390/s26020676

**Published:** 2026-01-20

**Authors:** William Girard, Haiping Xu, Donghui Yan

**Affiliations:** 1 Computer and Information Science Department, University of Massachusetts Dartmouth, Dartmouth, MA 02747, USA; wgirard@umassd.edu; 2 Mathematics Department, University of Massachusetts Dartmouth, Dartmouth, MA 02747, USA; dyan@umassd.edu

**Keywords:** marine visibility forecasting, self-adaptive deep learning, real-time training, time-series sensor data, online learning, maritime safety

## Abstract

Accurate prediction of marine visibility is critical for ensuring safe and efficient maritime operations, particularly in dynamic and data-sparse ocean environments. Although visibility reduction is a natural and unavoidable atmospheric phenomenon, improved short-term prediction can substantially enhance navigational safety and operational planning. While deep learning methods have demonstrated strong performance in land-based visibility prediction, their effectiveness in marine environments remains constrained by the lack of fixed observation stations, rapidly changing meteorological conditions, and pronounced spatiotemporal variability. This paper introduces SeADL, a self-adaptive deep learning framework for real-time marine visibility forecasting using multi-source time-series data from onboard sensors and drone-borne atmospheric measurements. SeADL incorporates a continuous online learning mechanism that updates model parameters in real time, enabling robust adaptation to both short-term weather fluctuations and long-term environmental trends. Case studies, including a realistic storm simulation, demonstrate that SeADL achieves high prediction accuracy and maintains robust performance under diverse and extreme conditions. These results highlight the potential of combining self-adaptive deep learning with real-time sensor streams to enhance marine situational awareness and improve operational safety in dynamic ocean environments.

## 1. Introduction

Weather forecasting plays a vital role in modern society, supporting safe and efficient transportation and operations on land, at sea, and in the air. Forecasting techniques have evolved substantially over time, from early instruments such as the mercury barometer in the seventeenth century to the widespread use of radar and satellite-based observation systems by the mid-twentieth century [1]. Despite these technological advances, operational forecasting remains an active and challenging area of research, particularly as climate change increases the frequency and intensity of extreme weather events, including heatwaves, heavy precipitation, non-tropical storms, and tropical cyclones [2]. Among the many variables involved in operational forecasting, visibility is one of the most critical. Visibility is commonly defined as the maximum horizontal distance at which an object can be clearly observed, and it directly influences both safety and operational efficiency. It is primarily governed by light extinction due to scattering and absorption by atmospheric constituents such as fog droplets, aerosols, precipitation, and clouds, as well as by radiative conditions related to solar elevation. Reduced visibility can limit airport capacity and disrupt flight schedules, increase the risk of road accidents, and significantly impair safety-critical operations in maritime environments [3,4,5]. In maritime settings in particular, low-visibility conditions are strongly associated with elevated risks of vessel collisions, navigational errors, and delayed or suspended search-and-rescue operations, especially in coastal and offshore regions [6]. Previous studies and incident analyses have identified fog and reduced visual range as contributing factors in a substantial fraction of maritime accidents [5], underscoring the importance of accurate visibility forecasting for safe navigation and effective emergency response. Beyond safety concerns, inaccurate visibility forecasts also impose significant economic costs through shipping delays, inefficient rerouting, and increased fuel consumption. Given that maritime transport accounts for more than 80% of global trade [7], even modest improvements in visibility forecasting accuracy can yield substantial operational and economic benefits. Reliable visibility prediction at sea is therefore both a scientific challenge and an operational necessity.

Recent advances in deep learning, particularly artificial neural networks (ANNs), have shown promising results for land-based visibility prediction [8,9]. Marine environments, however, present additional challenges. Oceanic conditions can change rapidly, and the open ocean lacks the dense networks of fixed observation stations commonly available on land. As a result, traditional ANN-based approaches often lack the adaptability required to perform reliably in dynamic and data-sparse marine settings. Although several approaches to marine visibility prediction have been explored, including fog nowcasting, image-based estimation, and numerical weather modeling systems [10,11,12], most existing methods remain limited in scope. They typically focus on short forecasting horizons, specific geographic regions, or particular weather phenomena. To the best of our knowledge, no existing framework has demonstrated robust, real-time marine visibility forecasting across diverse spatial and temporal scales. To address this gap, we introduce SeADL, a self-adaptive deep learning framework designed for reliable, real-time marine visibility forecasting under highly dynamic oceanic conditions. The proposed system employs a cluster of SeADL models that operate in parallel to generate visibility forecasts both at the vessel’s current location and at nearby remote locations. These timely predictions provide actionable decision support for ship captains and navigators, enabling safer routing, effective hazard avoidance, and informed operational planning. The input feature set is intentionally limited to conventional atmospheric variables and excludes explicit location and seasonal indicators. This design choice allows the models to adapt naturally to evolving temporal and spatial conditions through continuous online learning. Each model generates visibility forecasts at a predefined lead time for its assigned location. The remote locations are positioned approximately one mile from the vessel, supporting safe and practical deployment of drone-based atmospheric measurements.

This work extends our earlier real-time marine visibility forecasting framework introduced in [13]. In that study, a multilayer perceptron (MLP)-based deep neural network was used to estimate visibility under both normal and storm-related conditions. While effective, the MLP architecture treated meteorological observations as independent feature vectors and therefore did not explicitly capture temporal dependencies in the data. In the present study, we adopt Long Short-Term Memory (LSTM) networks [14], a specialized class of recurrent neural networks (RNNs) designed for sequence-based learning, to more accurately model the temporal evolution of marine weather conditions. By operating on temporally ordered input sequences, the LSTM-based SeADL models are able to represent both short-term fluctuations and longer-term visibility trends. This architectural shift, together with the proposed clustered modeling design, reflects the inherently sequential nature of meteorological processes and distinguishes the proposed framework from the snapshot-based approach used in [13]. The main contributions of this work are summarized as follows:

1. We propose a self-adaptive deep learning framework for real-time marine visibility forecasting that operates under dynamic and data-sparse oceanic conditions.
2. We introduce a clustered modeling architecture that simultaneously forecasts visibility at a vessel’s current location and at multiple nearby remote locations.
3. We develop an online, sequence-based training strategy using LSTM networks to capture both short-term fluctuations and longer-term visibility patterns.
4. We demonstrate the effectiveness of the proposed framework through realistic diurnal and storm-driven case studies.

The rest of the paper is organized as follows. Section 2 reviews related work relevant to marine visibility forecasting. Section 3 presents the problem formulation, data sources, and the proposed real-time visibility forecasting framework, including the LSTM-based SeADL models. Section 4 describes the time-series dataset design and the construction of the adaptive training minibatch. Section 5 details the selected hyperparameters and the algorithms used for real-time training and prediction. Section 6 presents the case studies, results, and discussion. Finally, Section 7 concludes the paper and outlines directions for future research.

## 2. Related Work

Visibility prediction has long been recognized as essential to transportation safety, motivating extensive research into models for detecting and forecasting low-visibility conditions and extreme weather events. Much of this progress has been driven by land-based studies, where dense observational networks and fixed weather stations provide continuous, high-quality measurements that support the development of sophisticated predictive models. For example, Abdel-Aty et al. investigated the risks associated with fog and smoke during nighttime driving and found that the early morning hours from December through February exhibit the highest likelihood of traffic accidents related to these conditions [15]. Jonnalagadda and Hashemi introduced an auto-regressive recurrent neural network (ARRNN) for land-based visibility prediction using weather data from multiple weather stations [8]. They showed that their approach outperforms standard RNNs and LSTMs in terms of the coefficient of determination (R^2^). Yang et al. developed a hybrid model combining LSTM and convolutional neural networks (CNNs) to predict extreme highway weather events [9]. Their predictions included fog and heavy rainfall, achieving nearly 90% prediction accuracy and clear improvements over traditional machine learning methods. Niu et al. used decision tree-based models, including XGBoost, LightGBM, and Random Forest, to forecast visibility across multiple weather stations and reported highly accurate short-term predictions [16]. In a related application, Dabhade et al. applied a ConvLSTM model for cyclone detection in India, reconstructing cyclone imagery and validating performance using PSNR and SSIM metrics [17]. These studies demonstrate the value of rich, station-based datasets for visibility prediction and extreme-weather detection in terrestrial environments. In contrast, our work focuses on the distinct and often more challenging conditions encountered in open-ocean settings. At sea, observational data are sparse, sensor coverage is limited, and weather conditions can change rapidly, making it difficult for traditional land-based models to perform reliably. To address these challenges, we propose a real-time, self-adaptive LSTM-based framework that continuously updates its parameters as new data become available. This adaptive design allows the model to track fast-changing conditions and produce accurate marine visibility forecasts, even during short-lived, rapidly developing storm events. Unlike previous studies that primarily emphasize detecting the onset of extreme events, our approach is designed to anticipate visibility changes before they occur, providing meaningful advantages for maritime navigation, operational planning, and overall safety at sea.

Advances in ocean observation technologies and the increasing availability of high-resolution environmental data have accelerated the shift toward data-driven approaches in marine forecasting. As a result, machine learning and deep learning techniques are now being explored in a wide range of oceanographic applications, from ecosystem monitoring to maritime operations. Muttil and Chau investigated algal bloom prediction using ANNs and genetic programming (GP) [18]. Their study highlighted the importance of feature ranking and suggested that the persistence observed in bloom dynamics may be linked to long flushing times in semi-enclosed coastal waters. Building on this trend, Kim et al. proposed a convolutional LSTM framework to forecast multiple ocean weather parameters, including sea surface temperature, wave height and period, wave direction, wind speed, and vessel speed [19]. Their results demonstrated that time-series image inputs can effectively capture spatiotemporal patterns and improve forecasting accuracy. In a related line of work, Krestenitis et al. employed deep convolutional neural networks (DCNNs) to detect oil spills from satellite imagery, illustrating the feasibility of computer vision techniques for large-scale environmental monitoring [20]. Similarly, Khoo et al. applied several CNN-based super-resolution models, including SRGAN, ESRGAN, and SN-ESRGAN, to enhance satellite-derived sea surface temperature data in the South China Sea, with SN-ESRGAN achieving the strongest performance [21]. These studies demonstrate how deep learning can improve the quality and utility of satellite-based ocean observations. More recently, Bakirci showed that the YOLOv9 detection algorithm can reliably identify vessels of varying sizes from satellite imagery, achieving a precision of 94.6% [22]. This work further underscores the growing role of advanced detection and recognition algorithms in maritime safety and surveillance. Despite this progress, many existing approaches either overlook temporal dependencies or limit their predictions to static or localized regions. For example, although Kim et al. [19] incorporated temporal information, their framework produced forecasts at relatively coarse 12 h intervals and across a limited number of sites. In contrast, our work introduces a fine-grained, real-time framework for forecasting marine visibility for a moving vessel. By integrating continuously updated onboard sensor measurements with drone-collected data, our approach supports adaptive, dynamic forecasting and offers practical benefits for maritime navigation and operational safety.

As real-time prediction becomes increasingly important in dynamic, data-rich environments, researchers have turned to models that can adapt continuously to rapidly changing conditions. These approaches aim not only to improve predictive accuracy but also to ensure that forecasts remain reliable, flexible, and responsive as new data arrive in real time. For example, Singhal and Ahmad developed a real-time facial recognition system for university attendance management [23]. By applying CNNs to live video streams, their system improved recognition accuracy while still meeting the speed requirements for real-time use. Similarly, Ford et al. introduced a real-time self-adaptive classifier (RT-SAC) for detecting suspicious bidders in online auctions [24]. By recognizing that bidding behavior can evolve quickly, their approach employed a moving-window strategy that enabled continuous model updates while maintaining strong detection performance. Real-time adaptability has also proven valuable in the energy sector. Girard et al. trained a deep learning model on a moving window of data to forecast hydropower generation [25], showing that real-time ANNs can produce accurate predictions across daily, weekly, and monthly time horizons for managing critical infrastructure. Building on these ideas, our prior work [13] extended real-time deep learning to the maritime domain by employing an MLP-based deep neural network to estimate visibility in the vicinity of a vessel. The present study expands this framework by introducing more diverse and realistic oceanic scenarios and by incorporating LSTM networks to more effectively capture temporal dependencies in visibility forecasting. This architectural enhancement results in a more robust and fine-grained real-time forecasting system tailored to the highly dynamic and uncertain conditions encountered in maritime navigation. Unlike many existing real-time systems that focus on static environments, single-task surveillance, or limited temporal contexts, the proposed approach operates aboard a moving maritime platform, integrates heterogeneous time-series data from onboard sensors and drones, and generates visibility forecasts simultaneously for both the vessel’s current location and nearby remote locations. Moreover, the framework establishes a foundation for future extensions, such as incorporating additional sensor modalities or embedding domain-specific physical constraints, which could further enhance adaptability and practical value for real-world maritime decision support.

## 3. Self-Adaptive Deep Learning for Marine Visibility Forecasting

### 3.1. Problem Formulation and Data Sources

Traditional deep learning models are typically designed for static or slowly changing prediction problems, such as estimating gross domestic product, recognizing faces, or diagnosing diseases. Maritime operations, however, take place in highly dynamic environments where visibility can degrade within minutes. In addition, reliable observational data are often scarce because oceanic weather stations provide limited spatial coverage. Thus, the combination of rapid environmental change and sparse data creates a fundamental challenge for accurate marine visibility forecasting.

In this study, marine visibility forecasting is formulated as a supervised regression problem. The objective is to predict a future visibility value at a predefined forecasting horizon *h* (e.g., 30 min ahead) based on recent meteorological observations. Visibility is treated as a continuous quantitative variable, measured in miles, and serves as the regression target throughout this study. At each time step, the model takes as input a temporally ordered sequence of multivariate atmospheric observations and produces a single continuous visibility prediction corresponding to the selected forecasting horizon. The forecasting horizon h represents the time interval between the most recent input observation and the future time point for which the prediction is generated. Let M denote a prediction model that maps a sequence of recent observations to a future visibility value. Formally, the problem of predicting future visibility v(t+h) is defined as in Equation (1).

(1) $$v(t+h)=M\left(X_{t}\right),\;X_{t}=(x_{t-\tau +1},\;\ldots \;,\;x_{t}),\;x_{k}\in \;R^{f}$$

where *τ* denotes the sequence length and *f* represents the number of input features. Each input sequence $X_{t}$ consists of τ consecutive feature vectors, with the most recent observation $x_{t}$. This formulation captures the temporal context required for forecasting future visibility at horizon *h*.

Because the proposed SeADL framework relies on data-driven learning, we first describe the data sources and input features used for real-time visibility forecasting. These inputs are drawn from onboard sensor measurements, drone-based observations, and derived environmental variables, and are combined into multivariate time-series sequences for model training and prediction. Marine visibility is governed by interacting thermodynamic, microphysical, and dynamical processes. Moisture-related variables influence fog and haze formation, wind conditions affect the transport and dispersion of aerosols and hydrometeors, and precipitation and cloud cover directly reduce visual range through scattering and attenuation of light. Solar elevation further affects perceived visibility during sunrise and sunset through changes in illumination and contrast. Careful feature selection is therefore critical in deep learning, since irrelevant or noisy inputs can introduce bias, reduce predictive accuracy, and increase computational cost [26,27,28]. In this study, the target variable is marine visibility, which can range from approximately 0.03 miles under dense fog to more than 27 miles under exceptionally clear conditions [29], with typical values between 10 and 15 miles. Accurate forecasting therefore requires input features that both reflect the underlying atmospheric processes and can be observed continuously during operation. Table 1 summarizes the selected input features used for real-time marine visibility forecasting. These features are obtained from onboard sensors, drone-based observations, or derived quantities computed from location and time. This design avoids reliance on satellite data, reducing data-collection complexity, latency, and dependence on external infrastructure, and thereby supporting real-time deployment in data-limited oceanic environments.

The selected features are grounded in well-established physical relationships with atmospheric visibility. Precipitation and cloud cover are directly associated with light scattering and attenuation, while temperature and dew-point temperature jointly determine humidity and saturation conditions critical to fog formation. Surface pressure provides context for large-scale weather systems and storm development. Wind speed and direction characterize the dispersion and transport of aerosols and moisture, as well as the movement of air masses and approaching weather systems. Observed visibility at the current time is included as a strong short-term predictor, particularly for near-term forecasting horizons. Finally, solar elevation and its sine and cosine representations capture diurnal radiative and illumination effects that strongly influence visibility transitions during sunrise and sunset. Meteorological variables at the vessel’s current location are collected using onboard sensors, while additional observations from surrounding regions are obtained via drone deployments or inferred through simulation based on local measurements. Drones equipped with specialized instruments, such as high-resolution cameras, can provide estimates of environmental conditions including cloud cover [30]. By integrating onboard measurements, drone-based observations, and derived parameters, the proposed framework addresses the challenge of sparse data availability in open-ocean environments while remaining suitable for real-time operation.

### 3.2. A Framework for Real-Time Visibility Prediction

The proposed real-time visibility prediction framework employs two types of SeADL models: one model corresponding to the vessel’s current location and four additional models representing nearby surrounding regions. Each model forecasts visibility over a fixed prediction horizon. The surrounding models generate predictions at locations approximately one mile from the vessel in the four cardinal directions (north, east, south, and west). These remote locations define a local safety zone that enables the system to anticipate deteriorating visibility conditions before the vessel encounters them. A predicted reduction in visibility in any direction serves as an early warning, allowing navigators to adjust course and avoid potentially hazardous areas. The one-mile offset provides sufficient lead time for course adjustments while remaining compatible with safe and practical drone-based data collection. These five models produce a spatially distributed representation of visibility that supports informed, weather-aware routing decisions in dynamic marine environments.

**Figure 1:**
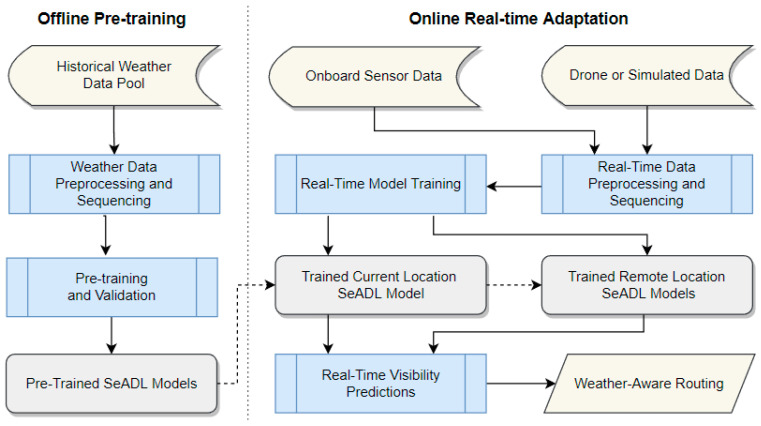
SeADL framework for real-time visibility prediction around a moving vessel.

Figure 1 presents an overview of the proposed framework, which operates in two stages: offline pre-training and online real-time adaptation. During the offline stage, SeADL models are pre-trained and validated using historical weather datasets to establish robust baseline performance and generalization across diverse environmental conditions. A fixed sequence length defines the temporal window used during both pre-training and online adaptation, ensuring consistency between historical learning and real-time updates. Model validation during offline training is performed using a held-out dataset for performance assessment and early stopping.

After deployment, the framework continuously ingests real-time observations from multiple sources, including onboard sensors, drone-based measurements, and simulated environmental estimates. These heterogeneous data streams are processed through a real-time preprocessing and sequencing pipeline before being used to incrementally update the models during operation. Because marine environments are inherently non-stationary, a fixed validation set is no longer appropriate during real-time operation. Instead, model performance is monitored using rolling prediction errors computed on newly observed data, which is standard practice in online and adaptive learning.

At initialization, the current-location and remote-location models share identical pre-trained parameters. As real-time operation proceeds, the models gradually diverge as they adapt to distinct streams of incoming observations. The current-location model is updated using direct sensor measurements collected aboard the vessel, while the remote-location models rely on drone observations or inferred environmental estimates to characterize visibility within the surrounding safety zone. This design allows each model to specialize according to its spatial context while maintaining coherence across the integrated visibility representation. All five models are updated online to generate rolling forecasts for both the vessel’s current position and nearby regions, directly supporting real-time navigational decision-making.

Positional information is incorporated implicitly rather than treated as an explicit input feature. Latitude and longitude are used to determine where observations are collected and how derived variables, such as solar elevation, are computed. This approach reduces the risk of overfitting to specific routes or regions and enables the models to generalize across different trajectories, seasons, and operating areas.

The framework operates in a vessel-centered moving reference frame rather than at fixed geographic locations. The current-location model is continuously anchored to the vessel’s instantaneous position, while the remote-location models are defined at fixed relative offsets with respect to the vessel. As the vessel moves, the geographic coordinates associated with all five models update continuously, ensuring spatial consistency between predictions and the surrounding marine environment.

The resulting predictions can be aggregated and visualized as time-series outputs describing the expected evolution of visibility across multiple forecasting horizons. In future implementations, these outputs may also be displayed through a live, radar-style visibility map, enabling operators to quickly identify hazardous regions and adjust routing strategies in real time. Unlike traditional static deep learning models, the proposed SeADL framework is inherently adaptive, supporting both short-term forecasting under rapidly changing conditions and longer-term trend analysis, such as diurnal or seasonal visibility patterns. This flexibility highlights the potential of the framework to support a wide range of maritime operational and decision-support applications.

### 3.3. Real-Time Updating of LSTM-Based SeADL Models

Since the proposed framework relies on modeling high-fidelity temporal dynamics, LSTM networks are a natural choice. These models are widely used in sequence-learning applications such as speech recognition and machine translation [31]. Unlike basic RNNs, which suffer from vanishing gradients and have difficulty retaining long-term information, they are specifically designed to capture temporal dependencies over long time horizons. Their gated-cell architecture, comprising input, forget, and output gates, controls how information is stored, discarded, and propagated, enabling stable memory retention across extended sequences [32]. This capability is particularly important for visibility forecasting, where observed conditions are influenced by both short-lived disturbances and longer-term temporal patterns.

**Figure 2:**
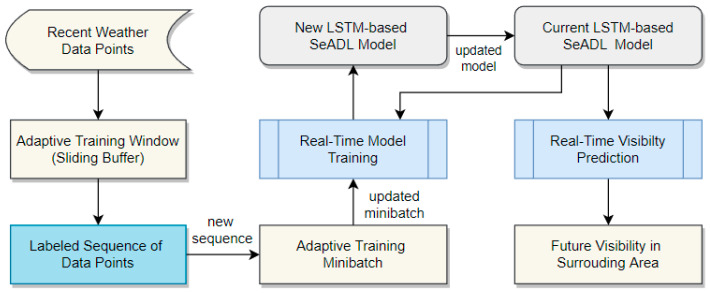
Overview of the real-time updating process for LSTM-based SeADL models.

The LSTM-based SeADL models are designed to operate in dynamic and rapidly changing maritime environments, where underlying data distributions may shift abruptly or drift gradually over time. Marine visibility exhibits both behaviors: storms can develop rapidly over open water, causing sudden visibility degradation, while diurnal cycles, seasonal variations, and longer-term climate trends introduce slower changes in the learning context. Relying solely on historical data for real-time prediction would therefore result in degraded performance as environmental conditions evolve and previously learned patterns become outdated. To address this challenge, the proposed framework adopts a minibatch-based, continuous training and evaluation strategy that allows the models to adapt incrementally to the most recent environmental observations. The overall real-time updating process for the LSTM-based SeADL models is illustrated in Figure 2.

As illustrated in Figure 2, the real-time updating process begins with the continuous collection of incoming weather data. These data populate an adaptive training window that operates as a sliding buffer: each new observation is added to the window while the oldest one is removed. Sequences constructed from this window are paired with continuous target values defined by the observed visibility at the forecasting horizon h. This process yields training examples that reflect the most recent temporal dynamics relevant to prediction. Preparing the data for LSTM training therefore involves both time shifting and sequence construction.

Each newly formed sequence is added to an adaptive training minibatch that retains only the most recent samples required for effective real-time training. The model is trained on overlapping sequences, with the sequence length determining the temporal context available to the LSTM. Longer sequences allow the SeADL model to capture extended meteorological trends, whereas shorter sequences emphasize rapidly evolving patterns associated with dynamic weather events. As new sequences enter the minibatch, older samples are discarded, enabling the model to continuously update its representation of current environmental conditions.

The updated minibatch is then passed to the real-time training module, where the SeADL model parameters are incrementally refined to produce an updated LSTM-based model for the next prediction cycle. Meanwhile, the current model is used to generate operational visibility forecasts. At each prediction step, an evaluation sequence is constructed from the most recent observations, and the model outputs a visibility estimate for the predefined forecasting horizon. These predictions provide timely insight into expected visibility conditions in the surrounding area and can be stored or visualized for decision-support purposes. At every time step, the newly trained model replaces the previous one, allowing the SeADL framework to adapt continuously to evolving marine conditions. This iterative cycle, comprising data collection, adaptive labeling, minibatch updating, incremental training, and online prediction, ensures that the system remains responsive to both sudden weather changes and gradual environmental shifts.

## 4. Time-Series Dataset Design and Adaptive Training Minibatch

### 4.1. Temporal Structuring of Time-Series Data

This study employs an LSTM-based architecture designed for time-series prediction and adaptive learning in evolving environments. LSTM networks are well suited to this task because they can model both short and long-term temporal dependencies while updating predictions continuously as new observations become available. This capability enables the proposed framework to respond quickly to emerging weather patterns while retaining contextual information from prior conditions, which is essential for real-time marine visibility forecasting. For LSTM-based forecasting to be effective, the input features must exhibit coherent and predictable temporal behavior. The variables selected for this study, summarized in Table 1, satisfy this requirement, as they evolve smoothly over time and follow well-understood physical processes. For example, air temperature typically decreases during nighttime hours as solar radiation diminishes. Observed visibility at time t is also included as an input feature, since it provides strong contextual information and is particularly informative for short-term visibility prediction. The datasets used in this study were generated to reflect smooth, physically plausible meteorological dynamics consistent with real marine environments.

LSTM models, like other RNNs, operate on sequences of observations and learn relationships between historical data and future outcomes. To support forecasting *h* minutes ahead, each input sequence $X_{t}$, whose most recent observation occurs at time t, is paired with the corresponding visibility value observed at time t+h, denoted as $v\left(t+h\right)$. This sequence-target pairing defines a supervised training example ${TE}_{t}$ and is formally expressed in Equation (2).

(2) $$TE_{t}=\left(X_{t},\;v\left(t+h\right)\right),\;X_{t}=\left(x_{t-\tau +1},\ldots ,x_{t}\right),\;x_{k}\in R^{f}$$

where *τ* denotes the sequence length and *f* represents the number of input features. Each sequence $X_{t}$ consists of τ temporally ordered feature vectors, ending with the most recent observation $x_{t}$. Each feature vector $x_{k}$ contains all selected input variables, including observed visibility, resulting in an input sequence of shape (*τ*, *f*). For computational efficiency, input sequences are processed in batches during training, yielding an input tensor of shape (*b*, *τ*, *f*), where *b* denotes the batch size. The sequence length *τ* and batch size *b* jointly influence the trade-off between capturing sufficient temporal context and maintaining model responsiveness. Accordingly, both parameters are treated as key hyperparameters in the SeADL models.

### 4.2. Adaptive Training Minibatch Construction for Real-Time LSTM Training

In this section, we describe the architecture and operation of the proposed adaptive training minibatch. The adaptive training minibatch is a compact, continuously updated collection of input sequences that includes both recently observed sequences used in prior training and newly constructed sequences derived from incoming data streams. This design enables continuous, incremental model adaptation while preserving sufficient recent historical context to ensure stable and reliable learning.

**Figure 3:**
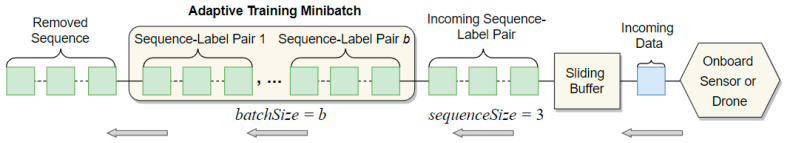
Example of an adaptive training minibatch composed of multiple input sequences.

Real-time training approaches based on moving training windows have proven effective in non-stationary environments [13,24,25]. In this work, we extend this concept to support multiple input sequences, making it suitable for training LSTM models that explicitly capture temporal dependencies. Rather than training on individual data points, incoming observations are organized into structured temporal sequences that are maintained within a dynamically updated minibatch. Figure 3 illustrates an example of an adaptive training minibatch with minibatch size *b* and sequence length 3.

In our prior work [13], the model was trained using a full data window in which each weather observation was paired with a corresponding visibility label. This formulation implicitly treats observations as independent, which is appropriate for feed-forward models but is not well suited for LSTM architectures that rely on temporal continuity. In contrast, the present work adopts a windowed minibatch of sequences, where each sequence contains *τ* temporally ordered observations. As shown in Figure 3, a sliding buffer of recent data points is maintained to construct training sequences. Using these sequences, training examples are formed as defined in Equation (2), ensuring that the LSTM model learns mappings from historical conditions to future visibility outcomes.

To further clarify the adaptive training process, we present Algorithm 1, which details the procedure for updating both the sliding buffer and the adaptive training minibatch. The algorithm supports continuous real-time training by incrementally incorporating newly observed data while retaining a limited set of recent training sequences.
**Algorithm 1.** Updating the Adaptive Training Minibatch**Input:** Most recent feature vector $x_{t}$ observed at time *t*, including visibility measurement *v*(*t*);  sequence length *τ*; minibatch size *b*; prediction horizon *h*; sliding buffer *B* **Output:** Updated adaptive training minibatch Γ1.  **if** sliding buffer *B* is empty **then**  // first invocation 2.   Initialize sliding buffer *B* using the most recent historical data points 3.   Initialize adaptive training minibatch Γ with *b* historical sequences of length *τ*,       each paired with its corresponding visibility value 4.  Append feature vector $x_{t}$ to the end of *B* 5.  Remove the oldest feature vector from *B* 6.  Let s←(t−h)//*s* is the most recent time for which the h-ahead label is now available 7.  Construct a training sequence $X_{s}$ from the oldest *τ* feature vectors in *B* 8.  Pair $X_{s}$ with visibility label $v\left(s+h\right)$ = *v*(*t*) // ($X_{s},\;v(t))$ is the latest sequence-label pair 9.  Append the new pair ($X_{s},\;v(t))$ to the end of Γ 10.   Remove the oldest sequence-label pair from Γ to maintain size *b* 11.   **return** updated Γ

At the beginning of real-time operation, Algorithm 1 initializes both the sliding buffer *B* and the adaptive training minibatch Γ using historical data to provide a stable starting point. At each subsequent time step *t*, the newly observed feature vector $x_{t}$, including the current visibility measurement *v*(*t*), is appended to *B*, while the oldest feature vector is discarded to maintain a fixed buffer length. The oldest *τ* feature vectors in *B* are then used to construct a training sequence whose most recent observation corresponds to time s=t−h. This sequence is paired with the visibility $v\left(s+h\right)=v(t)$, preserving the intended forecasting offset and temporal consistency between inputs and targets.

The resulting sequence-label pair is added to Γ, and the oldest pair is removed so that the minibatch remains fixed in size and focused on the most recent environmental conditions. The sliding buffer can also be used to construct an inference sequence by selecting the most recent τ feature vectors in *B*. This unified design enables a temporally consistent data representation for both real-time training and short-term forecasting.

### 4.3. Sequence Length and Minibatch Size for Diurnal and Storm Models

The choice of minibatch size involves an important trade-off between adaptability and temporal generalization. A small minibatch allows the model to fit closely to recent observations, thereby improving responsiveness to newly emerging patterns and abrupt environmental changes. However, such a configuration may limit the model’s ability to capture longer-term temporal dependencies and may increase sensitivity to short-term noise. Conversely, a larger minibatch incorporates a broader range of historical sequences, which can improve robustness to noise and facilitate the learning of more stable, long-term trends. In highly dynamic environments such as marine visibility forecasting, however, excessively large minibatches may reduce performance by slowing the model’s adaptation to rapidly and unpredictably changing conditions, which are often dominated by short-lived local effects.

Sequence length exhibits a similar trade-off. Longer sequences provide richer temporal context and allow the LSTM to model extended dependencies across time, which can be beneficial for slowly varying processes. However, they also increase computational cost and may hinder performance in volatile environments where recent observations are more informative than distant history. In addition, longer sequences substantially increase training time and memory requirements, making them less suitable for real-time learning scenarios in which timely model updates are critical. Building on prior work that conducted a detailed analysis of optimal window sizes [25], we performed a comprehensive set of experiments across multiple datasets to identify configurations best suited to our specific forecasting applications. Table 2 summarizes the tested and selected minibatch sizes and sequence lengths for both the diurnal and storm models.

It is important to note that the chosen configurations reflect the distinct temporal characteristics of the two forecasting applications. Slowly varying diurnal patterns favor longer sequences and larger minibatches to capture stable, long-term trends, whereas rapidly evolving storm conditions benefit from shorter sequences and smaller minibatches that support faster adaptation. This distinction arises from fundamental differences in temporal dynamics, rather than from whether the model is applied to local or remote sensing locations.

## 5. Real-Time Training of SeADL Models for Visibility Forecasting

### 5.1. Tuning of LSTM Hyperparameters

Hyperparameter selection plays a critical role in achieving accurate, stable, and responsive deep learning models. For the LSTM-based SeADL framework, key hyperparameters include the sequence length, minibatch size, number of neurons in each hidden layer, dropout rate, learning rate, optimizer, activation function, and number of training epochs. The selected sequence lengths and minibatch sizes for different application scenarios are summarized in Table 2. Table 3 presents the remaining hyperparameters shared across all SeADL configurations, together with the tested ranges and the final values selected for deployment. The maximum number of epochs and learning rate used during real-time training differ from those employed during offline pre-training in order to balance training stability with rapid adaptation to evolving environmental conditions. Together, Table 2 and Table 3 provide a comprehensive overview of the temporal and architectural design choices adopted in the SeADL framework. Most of the hyperparameter tuning reported in Table 3 was conducted using the GridSearchCV utility from scikit-learn, which systematically evaluates combinations of candidate values to identify robust configurations. This automated search was guided by validation performance and stability considerations to reduce overfitting while ensuring reliable convergence. In contrast, the maximum numbers of epochs used during pre-training and real-time training were selected through manual experimentation, as these parameters directly influence convergence speed and stability during online model updates. To maintain computational feasibility, only a representative subset of the hyperparameter search space was explored.

### 5.2. SeADL Model Pre-Training

The SeADL models are implemented as LSTM networks using the Keras API built on TensorFlow, which provides high-level abstractions for constructing sequential models with recurrent layers. Pre-training on a sufficiently large historical dataset is a crucial step, as prior studies have shown that effective pre-training substantially improves prediction accuracy, convergence stability, and robustness during subsequent online adaptation [13]. Specifically, pre-training enables the model to capture general temporal structures, seasonal trends, and baseline relationships before encountering the non-stationary and rapidly evolving conditions present during real-time operation. This initialization reduces sensitivity to noise and mitigates unstable parameter updates during early stages of online learning. During pre-training, the models are optimized using mean squared error (MSE) as the primary loss function, while mean absolute percentage error (MAPE) is used as an auxiliary evaluation metric. These metrics are defined in Equations (3) and (4).

(3) $$MSE=\frac{1}{n}\sum_{i=1}^{n}{\left(y_{i}-{\hat{y}}_{i}\right)}^{2}$$

(4) $$MAPE=\frac{100}{n}\sum_{i=1}^{n}\left|\frac{y_{i}-{\hat{y}}_{i}}{max(y_{i},\epsilon )}\right|,\;\epsilon >0$$

where $y_{i}$ and ${\hat{y}}_{i}$ denote the ground-truth and predicted values at time step i, respectively, and n is the total number of samples. MSE emphasizes larger prediction errors and therefore guides stable optimization during training. MAPE measures the relative prediction error as a percentage, making it scale-independent and easily interpretable across diverse operating conditions. The inclusion of the small positive constant ϵ in Equation (4) ensures numerical stability when $y_{i}$ approaches zero. This safeguard prevents division by zero and avoids disproportionately large error values, which commonly arise in dense fog conditions during visibility forecasting. By using $max(y_{i},\epsilon )$, the metric preserves the relative-error interpretation of MAPE while remaining well defined for all samples. Lower MAPE values indicate higher predictive accuracy, with zero corresponding to perfect predictions. Note that MAPE is unbounded and may exceed 100% when prediction errors are larger than the corresponding ground-truth values, particularly when true values are small.

**Figure 4:**
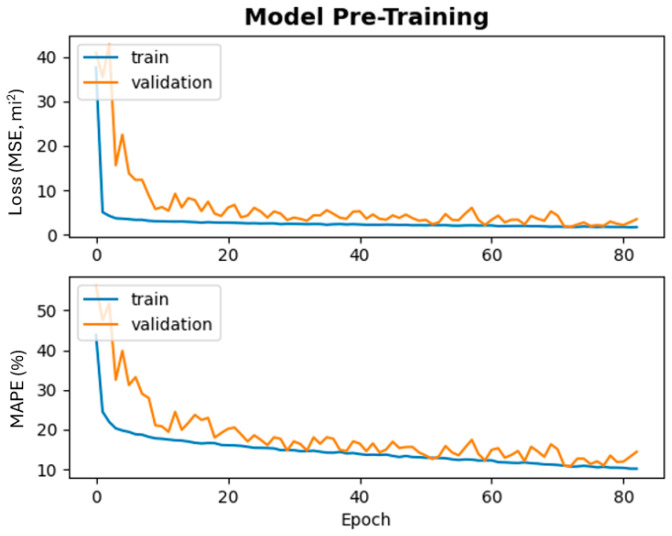
Pre-training MSE loss and MAPE curves for a SeADL model.

The SeADL models are pre-trained using Keras’s *fit* function with a standardized 20% validation split. Because the data are time-series in nature, all training and validation samples remain unshuffled to preserve temporal ordering. During pre-training, the *EarlyStopping* utility provided by Keras is employed to terminate training once the validation loss plateaus, thereby reducing overfitting and unnecessary computation. Early stopping is triggered based on validation loss trends rather than training loss to ensure robust generalization performance. Figure 4 illustrates the pre-training results for the diurnal current-location SeADL model, with the top panel showing MSE loss curves and the bottom panel showing corresponding MAPE values. Although the maximum number of pre-training epochs is set to 100, the figure shows that *EarlyStopping* typically halts training after approximately 80 epochs, indicating stable convergence well before reaching the predefined limit.

As shown in Figure 4, the training and validation curves initially exhibit noticeable separation, reflecting early-stage model adjustment to the underlying data distribution. As training progresses, both MSE and MAPE decrease steadily and the curves gradually converge. By the end of pre-training, the metrics stabilize at relatively low values, and the training and validation curves closely align, indicating effective convergence and strong generalization performance. These results confirm that the model has successfully learned meaningful temporal patterns from historical data and is well prepared for subsequent real-time adaptation.

### 5.3. Real-Time Training of SeADL and Visibility Forecasting

In rapidly evolving environments such as weather forecasting and online auctions, traditional offline deep learning approaches are often impractical. These methods typically assume stationary data distributions and require costly retraining to maintain performance. In contrast, deep learning models that support incremental fine-tuning can adapt continuously, enabling sustained predictive accuracy in non-stationary settings. In the context of marine weather forecasting, the proposed SeADL framework is specifically designed to capture both long-term trends (e.g., gradual, climate-driven changes in visibility) and short-term dynamics (e.g., rapidly developing storm events) through continuous real-time adaptation.

In the proposed architecture, the current-location SeADL model primarily learns diurnal visibility patterns, while four remote-location SeADL models focus on storm-driven visibility forecasting. All five models operate concurrently, with each independently generating visibility forecasts for the next *h* minutes at its assigned location. This parallel design provides a comprehensive and timely view of surrounding marine environment. When needed, additional SeADL models can be deployed to support longer-term forecasting objectives, such as climate-scale visibility analysis.

To quantitatively evaluate real-time model adaptation, we define an online training accuracy metric, denoted by ${Accu}_{training}$, as shown in Equation (5).

(5) $${Accu}_{training}=max(0,\;100-MAPE)$$

This formulation constrains training accuracy to the range [0, 100] and ensures that it increases monotonically as predictive performance improves. Derived directly from MAPE, this metric provides a more intuitive and interpretable measure of model quality during real-time updates.

Algorithm 2 outlines the real-time training and prediction workflow for a single SeADL model Φ, illustrating how adaptive fine-tuning and short-horizon visibility forecasting are tightly integrated in an online setting. The algorithm runs continuously as new sensor observations arrive, allowing the model to update incrementally while simultaneously producing forecasts without interrupting real-time operation.
**Algorithm 2.** Real-Time Training and Prediction for a Single SeADL Model**Input:** SeADL model Φ with sequence length *τ*, minibatch size *b*, and maximum minibatch epochs $e_{max}$; prediction horizon *h*; sampling rate *r* (where *r* divides *h*) **Output:** Updated SeADL model Φ and predicted visibility $v\left(t+h\right)$
1.  Compute prediction offset pos ←h/r 2.  **if** sliding buffer *B* does not exist **then** 3.    Create an empty buffer *B* of size *τ* + *pos* for Φ 4.  Collect features $x_{t}$ from Φ’s active location (current or remote) 5.  Invoke Algorithm 1 to construct an adaptive training minibatch Γ_Φ_ with |Γ_Φ_ | = *b* 6.  Initialize epoch count e ←0 and previous average training accuracy ${Accu}_{pre}\leftarrow 0$ 7.  **while** ${e<e}_{max}$ **do** 8.    Initialize accumulated training accuracy ${Accu}_{sum}\leftarrow 0$ 9.    **for** each sequence-label pair *α* in Γ_Φ_ **do** 10.     Fine-tune model Φ on *α* using backpropagation 11.     Compute training accuracy ${Accu}_{training}$ using Equation (5) 12.     ${Accu}_{sum}\leftarrow \;{Accu}_{sum}+{Accu}_{training}$ 13.   Compute average training accuracy ${Accu}_{avg}\leftarrow \;{Accu}_{sum}/b$ 14.   **if** ${Accu}_{avg}\leq {Accu}_{pre}$ and ${Accu}_{avg}>95$ **then break** 15.   ${Accu}_{pre}\leftarrow \;{Accu}_{avg}$; e←e+1 16. Extract prediction sequence $X_{t}$ of length *τ* from *B* 17. Generate a visibility forecast $v\left(t+h\right)$ for Φ’s active location 18. **return** updated SeADL model Φ and predicted visibility $v\left(t+h\right)$


Algorithm 2 begins by computing the prediction offset pos = h/r, which converts the prediction horizon from physical time into discrete sampling steps. For example, when the forecasting horizon is 30 min and sensor data are collected every 5 min, *pos* = 6. An empty sliding buffer B of length τ+pos is then created, if not already present, to store recent feature vectors. This buffer ensures correct temporal alignment between input sequences and future visibility labels and supports both training and prediction using a unified data structure.

At each time step, the most recent feature vector $x_{t}$ is collected from model Φ’s assigned location, and Algorithm 1 is invoked to construct an adaptive training minibatch Γ_Φ_ that incorporates the latest observations. The training loop then performs incremental fine-tuning for up to $e_{max}$ epochs using backpropagation. During each epoch, training accuracy is accumulated across all minibatch samples and averaged. To balance rapid adaptation with training stability, an early-stopping criterion is applied. Specifically, fine-tuning terminates when average training accuracy no longer improves and has already exceeded a high-confidence threshold of 95%. This mechanism prevents unnecessary updates once the model has sufficiently adapted. It also reduces the risk of overfitting and catastrophic forgetting of previously learned temporal patterns.

Following the training phase, the most recent τ feature vectors in buffer *B* are used to construct the input sequence $X_{t}$, which is then passed to the model to generate a visibility forecast v(t+h). The algorithm returns both the updated SeADL model and the predicted visibility, enabling downstream tasks such as visualization, decision support, and higher-level planning.

For simplicity, Algorithm 2 assumes that the training and prediction intervals coincide with the data sampling interval. In practice, these intervals can be configured independently. Less frequent training reduces computational overhead and mitigates overfitting, whereas more frequent updates are beneficial in highly dynamic marine environments, particularly during severe storm events. Similarly, longer prediction horizons may be sufficient under stable conditions, while shorter horizons are preferable during periods of rapid weather change. Finally, each SeADL model can be executed in a dedicated thread, allowing models associated with the current location and remote locations to operate concurrently. This parallel execution strategy improves computational efficiency and supports scalable real-time deployment of the proposed SeADL framework in multi-location marine monitoring scenarios.

## 6. Case Studies

To demonstrate the feasibility and effectiveness of the proposed framework, we present a series of representative case studies conducted under realistic maritime operating conditions. First, we evaluate the current-location SeADL model by examining its ability to capture long-term temporal patterns in visibility, focusing on day-night cycles observed over multiple months as seasonal changes gradually shift sunrise and sunset times. Next, we assess the predictive performance of a remote-location SeADL model in forecasting visibility during a simulated storm event. Finally, we simulate a realistic ship navigation scenario around a developing storm to illustrate the practical applicability and real-time decision-support capabilities of the proposed approach.

### 6.1. Experimental Setup and Dataset Descriptions

The weather-related features used for sea-based visibility prediction are summarized in Table 1. Several features, such as solar elevation, are computed directly from the vessel’s latitude and longitude. Other variables, including visibility, are obtained from the International Comprehensive Ocean-Atmosphere Data Set (ICOADS) through NOAA’s data portal [29]. Cloud cover and precipitation data are sourced from the Copernicus Climate Data Store, which provides global gridded daily and monthly observations from 1979 to the present [33]. Although cloud cover and precipitation are derived from satellite observations, these features can alternatively be obtained from onboard sensors in real-world deployments, reducing reliance on external data sources. All datasets are cleaned and preprocessed using the Pandas library in Python (Python 3.12), including the removal of missing values and irrelevant attributes. The spatial domain is restricted to an offshore region near South Carolina, USA (30–36° latitude, −80° to −70° longitude).

In operational maritime settings, shipboard sensors can collect weather observations at high temporal resolution, such as every five minutes. In contrast, datasets such as ICOADS and Copernicus provide daily or sparsely sampled measurements. ICOADS consists of irregularly distributed ship-based point observations with temporal resolution ranging from sub-daily to daily; in this study, it is used at a daily resolution to establish baseline visibility and meteorological conditions near the vessel’s location. These baseline values do not directly align with high-frequency onboard sensor data and therefore serve only as initialization and contextual reference. High-frequency (five-minute) observations used for real-time training and prediction are obtained from onboard sensors, drone-based measurements, or simulated time series aligned with the vessel’s trajectory. To bridge the temporal gap between baseline and real-time data, intermediate observations are generated to construct realistic five-minute time series.

Under diurnal conditions, visibility typically increases after sunrise, stabilizes around midday, decreases at sunset, and remains low overnight. For example, under clear nighttime conditions in the open ocean, visibility may be approximately 2.8 miles [34]. Because ICOADS provides daily average visibility values, nighttime visibility is estimated as a fraction of the daytime baseline. To enhance realism, controlled Gaussian noise is added to the simulated visibility profiles.

The remaining meteorological features are generated following established simulation methodologies [35,36], using deterministic seasonal trends combined with stochastic residual components. Many atmospheric variables exhibit predictable temporal behavior; for instance, temperature often peaks near midday and reaches its minimum around midnight. Accordingly, deterministic trends are first derived from daily mean values, after which controlled randomness is introduced to produce realistic five-minute observations. Storm datasets are generated using a similar approach. To incorporate realistic storm dynamics, wind speed and pressure profiles are obtained from NOAA’s Historical Hurricane Tracks database [37], and visibility during storm events is simulated using distance-based attenuation functions that account for the vessel’s proximity to the storm.

Data from onboard sensors, drone-based observations, and simulated estimates are integrated through temporal synchronization and spatial association. All measurements are resampled or generated at a common temporal resolution and aligned to a shared time index. Spatially, each measurement is associated with either the vessel’s current position or a predefined relative offset. Using the synchronized and spatially aligned measurements described above, two datasets are constructed. A diurnal dataset is generated for the current-location model, spanning 1 February to 31 May 2019, to evaluate adaptation to seasonal shifts in sunrise and sunset times. This dataset contains 34,560 five-minute observations. In addition, a storm-scenario dataset is created for visibility prediction under adverse conditions. It consists of 60 simulated, non-tropical storm events with varying durations and intensities, each represented as a localized weather system relative to the vessel and sampled at five-minute intervals. Baseline meteorological variables are obtained from ICOADS, Copernicus, and NOAA historical non-tropical storm and severe weather archives, while high-frequency dynamics are generated through interpolation and controlled stochastic perturbations. Severe hurricanes are not considered, as they are typically detected well in advance by operational radar systems. Instead, the simulated storms represent localized, non-tropical weather systems that do not meet the meteorological definitions of tropical depressions, tropical storms, or hurricanes, as defined by the National Weather Service [38]. The resulting storm-scenario dataset contains 4084 five-minute observations and is designed to evaluate the robustness and adaptability of the proposed framework under rapidly changing conditions. All experiments employ a 30 min forecasting horizon and are conducted on a workstation equipped with 16 GB of RAM, an Intel Core i5-12400F CPU, and an NVIDIA GeForce RTX 3060 GPU with 12 GB of VRAM.

### 6.2. Normal Weather with Day-Night Cycle

The current-location SeADL model was evaluated using day-night visibility data spanning from March 1 to May 31, 2019. Prior to real-time evaluation, the model was pre-trained on data from February to provide a stable initialization and ensure reliable predictive performance at the start of the real-time training process. Evaluating the model over multiple months allows us to examine its ability to adapt to gradual seasonal changes, including shifts in sunrise and sunset times. To reduce the risk of overfitting, real-time training was performed at three-hour intervals. Table 4 reports the monthly training accuracy and the mean absolute error (MAE), as defined in Equation (5) and Equation (6), respectively.

(6) $$MAE=\frac{1}{n}\sum_{i=1}^{n}|y_{i}-{\hat{y}}_{i}|$$

where $y_{i}$ and ${\hat{y}}_{i}$ denote the ground-truth and predicted values at time step i, respectively, and n is the total number of samples. The MAE measures the average magnitude of the prediction error in the original visibility units, providing an intuitive and interpretable indicator of forecasting accuracy.

As shown in Table 4, the model consistently achieves strong performance, with all monthly accuracies exceeding 94.9% and an overall average accuracy of 95.46%. The MAE remains low throughout the evaluation period, averaging 0.246 miles across the three months. Importantly, model performance does not degrade over time; instead, it improves as real-time training progresses. In particular, May exhibits both the highest accuracy and the lowest MAE. This improvement can be attributed to two primary factors: (1) the cumulative effect of extended real-time training, which continuously refines the model parameters, and (2) the generally more stable visibility conditions observed in May compared with earlier months. For comparison, the same model was evaluated without real-time training. Under this setting, the average accuracy decreased to 81.07%, while the MAE increased to 1.32 miles. Thus, the incorporation of real-time training improved accuracy by 14.39% and reduced MAE by 1.07 miles, providing strong evidence of the effectiveness of the proposed adaptive learning mechanism.

**Figure 5:**
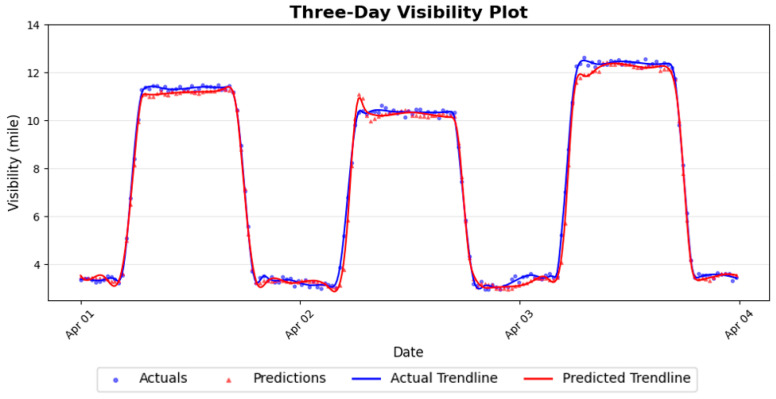
Real-time visibility prediction during the first three days in April (every 5th data point).

To further assess real-world operational performance, we evaluated the model using a continuous stream of sensor data over several consecutive days. Figure 5 illustrates the real-time visibility predictions during the first three days of April.

As illustrated in Figure 5, the predicted visibility closely follows the observed values despite substantial day-to-day variability. The model accurately captures the diurnal visibility cycle, including nighttime reductions and post-sunrise recovery, which are among the most challenging periods due to rapid environmental changes. During periods of abrupt visibility change, the model’s predictions remain closely aligned with the ground truth, with minimal lag or overshoot. These results indicate that the model effectively captures both short-term variations and longer-term temporal patterns.

**Figure 6:**
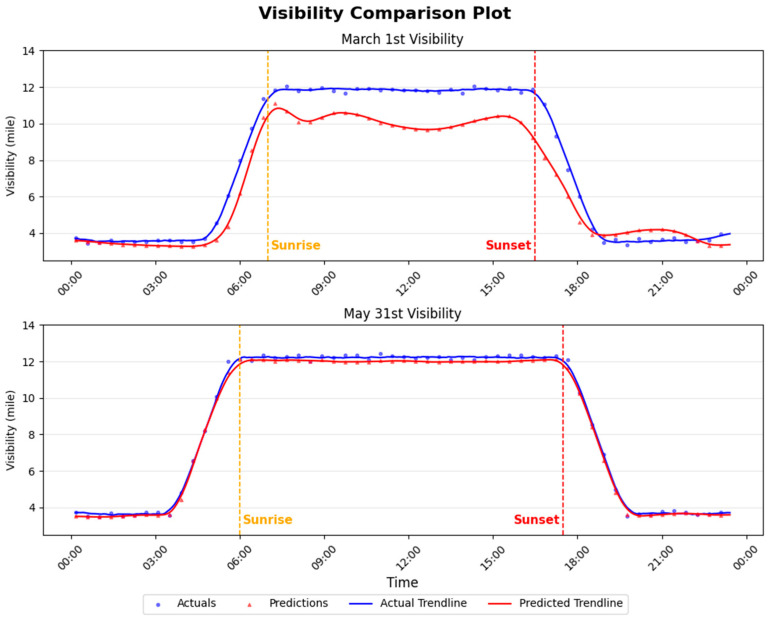
Visibility comparison between 1 March and 31 May (every 5th data point). The daylight duration increases from 9 h and 30 min on 1 March to 11 h and 30 min on 31 May.

A key advantage of real-time training is its ability to gradually adapt to long-term trends driven by seasonal variability or broader climatic influences. Unlike static models trained solely on historical data, a continuously updated model can adjust its internal parameters as new and evolving environmental patterns emerge. While Figure 5 demonstrates the model’s ability to track visibility trends over several consecutive days, it does not fully capture how performance evolves over extended periods or how the model responds to systematic environmental shifts. To highlight these long-term adaptation effects, we focus on the gradual changes in sunrise and sunset times over the spring season. Figure 6 compares model performance on the first day of real-time training, 1 March, with that on the final day, 31 May.

As shown in Figure 6, the top subplot compares the actual and predicted visibility on 1 March, while the bottom subplot presents the corresponding comparison for 31 May. Vertical dashed lines indicate the times of sunrise and sunset. The predictions on 1 March exhibit greater variability and noise, reflecting the model’s early stage of adaptation. In contrast, the predictions on 31 May align much more closely with the observed visibility. Despite a 30 min forecasting horizon, the 31 May predictions show little to no observable delay. This improvement clearly demonstrates the benefit of continuous real-time training, which progressively refines the model and significantly reduces prediction error over time.

### 6.3. Forecasting Visibility Under a Simulated Storm Scenario

While overall accuracy and long-term performance are important, challenging scenarios are particularly valuable for evaluating a model’s robustness under rapidly changing conditions. Such scenarios reveal how effectively a model responds to abrupt environmental changes and whether its learned representations remain reliable during adverse weather. In this study, we examine a simulated storm scenario involving a developing storm located approximately five miles north of the vessel. Storms of this type are commonly associated with strong winds, heavy precipitation, dense cloud cover, and low atmospheric pressure, all of which pose significant challenges for visibility forecasting.

Environmental measurements near the vessel are assumed to be collected using a remote drone. To balance data quality with operational safety, the drone is positioned approximately one mile from the vessel, reducing the risk of equipment loss while still capturing representative atmospheric and visibility conditions. This remote vantage point enables ship operators to assess visibility gradients around the storm. In particular, a declining visibility trend in a given direction may indicate the need to adjust course to avoid hazardous conditions. Because the definition of a safe operating zone depends on navigator judgment and operational context, we adopt a visibility threshold of five miles as an indicative safety criterion. This threshold corresponds to the visibility measured at the remote drone location; visibility at the vessel itself would typically be higher, depending on the storm’s movement and intensity.

SeADL is formulated as a regression model that predicts continuous visibility values. Accordingly, operational interpretations such as “safe” or “unsafe” conditions are inherently application-dependent and rely on visibility thresholds defined by navigational practice. In this study, such terms are used illustratively to aid interpretation rather than to imply a formal classification framework.

**Figure 7:**
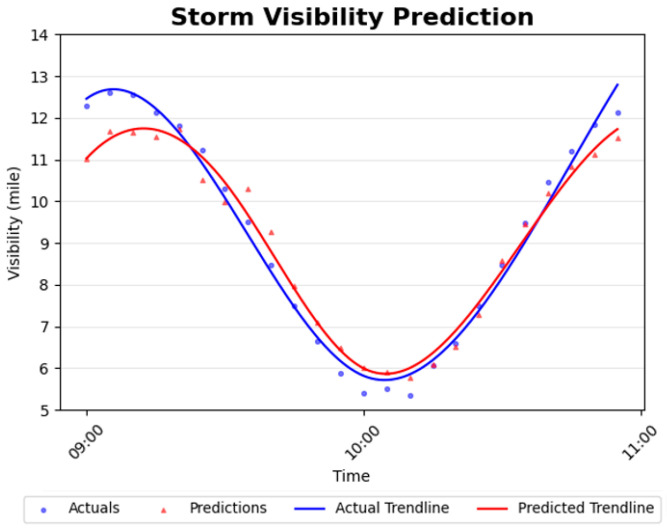
Real-time prediction of a simulated storm at a remote location.

After offline pre-training and real-time adaptation on storm-related data, we evaluate the SeADL model using a 120 min simulated storm event to assess predictive performance under rapidly evolving conditions. Figure 7 presents the real-time prediction results for the north-direction remote-location model using a 30 min forecasting horizon. In this scenario, the remote drone is positioned approximately four miles from the storm center and therefore does not experience the storm at full intensity.

As shown in Figure 7, visibility decreases sharply at storm onset, reaches a minimum around 10:00 AM, and then gradually recovers as the storm dissipates. While the model closely tracks overall visibility trends, it occasionally overestimates visibility during the most severe low-visibility intervals. This behavior highlights the difficulty of forecasting rare extreme conditions under limited training data and motivates future work focused on low-visibility–aware training and evaluation.

To account for the high variability of real-world marine environments, we introduce controlled stochasticity by adding Gaussian noise to the deterministic weather backbones, as described in Section 6.1. Zero-mean Gaussian noise is generated using the numpy.random.normal function, with the standard deviation (STD) explicitly controlling the magnitude of variability. Smaller standard deviations introduce only minor perturbations that preserve underlying temporal trends, whereas larger standard deviations produce increasingly pronounced deviations. Table 5 summarizes the sensitivity analysis across six noise levels, reporting both MAPE and prediction accuracy, defined consistently with the training accuracy in Equation (5). All experiments are conducted using the two-hour simulated storm dataset.

As anticipated, both MAPE and prediction accuracy degrade as noise levels increase. When the standard deviation is small, features closely follow the underlying storm trend, allowing the model to maintain low regression error and high accuracy. In contrast, at very high noise levels (STD = 2.0), visibility values may fluctuate rapidly, for example, ranging from approximately 2.6 to 5.5 miles within a five-minute interval. Such extreme variability introduces substantial unpredictability, which naturally increases forecasting error and reduces overall accuracy.

Finally, it is important to note that the simulated scenario represents partial exposure to storm-influenced conditions rather than passage through a fully developed storm core. In this setup, the vessel operates near, but not within, the storm center, reflecting a realistic operational situation in which navigators intentionally maintain a safe distance from severe weather. As a result, the framework focuses on evaluating visibility gradients and anticipating deteriorating conditions as the vessel approaches adverse environments, rather than modeling direct storm-core encounters. Forecasting visibility within fully developed storm cores is therefore beyond the scope of the present study, but it remains an important direction for future research.

### 6.4. Visibility-Based Navigation of a Moving Vessel Around a Nearby Storm

In this final case study, we investigate visibility-aware navigation for a vessel maneuvering in the vicinity of a developing storm. To this end, we employ four remote-location models to forecast the spatiotemporal evolution of visibility as the vessel progresses along its route. Specifically, drones are deployed to the north, east, south, and west of the vessel, enabling continuous monitoring of visibility conditions in all directions and supporting more informed navigation decisions.

For this scenario, we assume a predetermined vessel trajectory that passes along the eastern (right-hand) side of the storm. Consistent with the previous case study, the remote locations do not intersect the storm’s core but instead experience progressively weaker, storm-like conditions as their distance to the storm increases. Accurately quantifying visibility degradation as a function of distance from the storm cell is nontrivial and requires careful treatment to ensure physically meaningful values. To address this challenge, we introduce a visibility-reduction model based on an exponential decay formulation. The distance-dependent scaling function g(r) and the resulting visibility function V(r,t) are defined in Equations (7) and (8), respectively.

(7) $$g\left(r\right)=\left\{\begin{array}{cc} 1, & r\leq R_{core}\\ exp\left(-\frac{r-R_{core}}{L}\right), & r>R_{core} \end{array}\right.$$

(8) $$V\left(r,\;t\right)=V_{0}-\left(V_{0}-V_{c}\left(t\right)\right)\;g(r)$$

where r denotes the instantaneous distance from the storm center, $R_{core}$ is the storm-core radius, $V_{0}$ represents the nominal clear-weather visibility, L is the decay length scale, and $V_{c}(t)$ is the storm-core visibility at time t. When the vessel is located within or at the boundary of the storm core ($r\leq R_{core}$), the scaling factor equals one, forcing the vessel’s visibility to match that of the storm. As the distance from the storm increases, the scaling factor decays exponentially, rapidly diminishing the storm’s influence on visibility. Beyond a sufficiently large distance (approximately 12 miles in this study for non-tropical storms), the storm’s impact becomes negligible.

Equations (7) and (8) define a phenomenological visibility-reduction model that approximates the degradation of horizontal visibility as a function of a vessel’s distance from a storm center. The formulation is motivated by empirical observations that precipitation intensity, cloud density, and aerosol concentration generally increase in proximity to storm centers, leading to reduced visibility. While storms do not universally degrade visibility under all conditions, this model captures a representative scenario commonly encountered in maritime environments and provides a controlled mechanism for evaluating adaptive learning behavior under adverse conditions. Similar distance-based degradation formulations have been adopted in atmospheric and environmental modeling, where storm-related or visibility-related parameters are incorporated into distance-dependent equations to represent reduced environmental conditions during storm events and tropical cyclones [39,40]. This supports the plausibility of adopting a distance-dependent visibility-reduction formulation in our model.

In our simulations, we set the storm-core radius to $R_{core}=3$ miles and define the storm’s effective influence region to extend to 12 miles. Accordingly, once r=12, the storm contributes minimally to the observed visibility. We further set $V_{0}=12$ miles, corresponding to clear-weather visibility under typical sunny conditions, consistent with NOAA observations. Enforcing a residual storm influence of 5% at a distance of r=12 miles yields Equation (9).

(9) $$g\left(12\right)=exp\left(-\frac{12-R_{core}}{L}\right)=0.05$$

**Figure 8:**
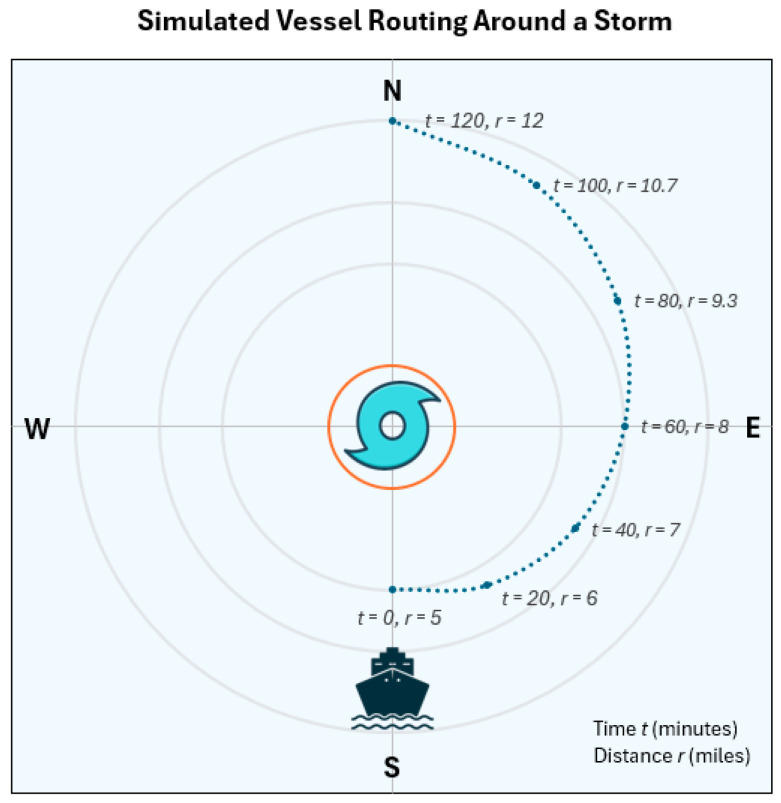
Simulated vessel trajectory around a stationary storm. The red circle at the center denotes the storm cell with a radius of three miles, while the navy-blue dotted curve represents the vessel’s visibility-aware trajectory.

Solving Equation (9) gives L≈3.00. With this visibility-attenuation model established and its parameters fully specified, we present the simulated vessel trajectory around a stationary storm in Figure 8. The vessel initially travels close to the storm, reflecting the assumption that the storm’s development is not known a priori to the vessel. As the simulation progresses, the vessel gradually increases its distance from the storm before ultimately returning to its original planned route toward the north. Each data point along the trajectory is annotated with the corresponding time t (in minutes) and distance from the storm center r (in miles).

In this case study, we assume a time-varying, parabolic visibility profile for the storm. The storm-core visibility, $V_{c}(t)$, starts at 12 miles when t=0 min, decreases to a minimum of 3 miles at t=60 min, and then gradually recovers to 12 miles by t=120 min. Because high-resolution storm visibility data at five-minute intervals were unavailable, this profile was constructed using real storm observations from NOAA’s Storm Events Database as a guiding reference. As shown in Figure 8, when the storm reaches its lowest visibility at t=60 min, the vessel is located approximately 8 miles from the storm center, resulting in an estimated vessel visibility of about 10.3 miles. Consequently, the minimum visibility experienced by the vessel along the entire trajectory remains approximately 10.3 miles, which is well within typical safe operating conditions for maritime navigation.

**Figure 9:**
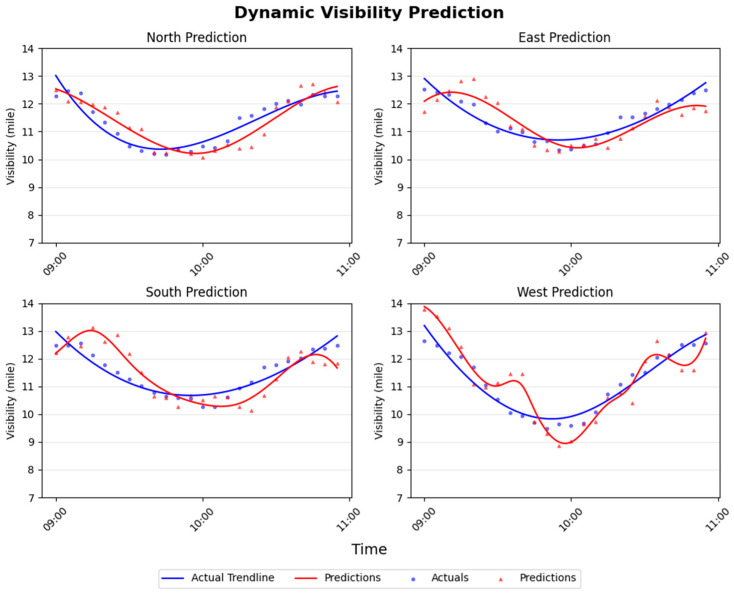
Real-time visibility prediction during vessel navigation around a storm.

Aligning with the vessel trajectory and simulated storm conditions shown in Figure 8 and Figure 9 further presents the corresponding true and predicted visibility values for all four directions. A dedicated predictive model is employed for each direction, enabling a comprehensive evaluation of directional visibility forecasting performance. The most pronounced visibility reductions occur between approximately 9:40 and 10:00 AM, corresponding to roughly one hour into the vessel’s journey. Throughout the navigation, the north, south, and east directions maintain relatively high visibility levels, while the west direction exhibits the most significant degradation due to its closer proximity to the storm. Nevertheless, visibility in the west direction remains above 9 miles at all times, indicating that the vessel does not encounter critically low-visibility conditions. By the end of the maneuver, visibility in all four directions returns to high levels, confirming a successful traversal around the storm. The corresponding remote-direction models demonstrate strong predictive performance, with only minor timing offsets or small deviations from the ground truth. Even the largest prediction error across all models remains within approximately one mile. This simplified scenario highlights the practical value of integrating multiple SeADL models operating across different spatial directions, as they provide a robust situational awareness framework that can support safer and more effective navigation decisions in potentially hazardous maritime environments.

### 6.5. Discussion

At present, publicly available, high-frequency, long-duration visibility datasets for the open ocean remain extremely limited. This lack of standardized, benchmark-quality data constrains direct, quantitative comparison with existing maritime forecasting systems or learning-based baselines under identical operational conditions. The datasets used in this study combine observations from multiple sources that differ in spatial resolution, temporal sampling rate, and measurement precision. While established observational repositories provide daily or sparsely sampled measurements, high-frequency (five-minute) time series are generated through interpolation and controlled stochastic perturbations to better reflect short-term variability. This approach represents a practical compromise motivated by the lack of long-duration, high-frequency visibility measurements over the open ocean. As a result, the simulated data cannot fully reproduce the complex and chaotic variability of real marine environments, particularly at fine spatial and temporal scales. Accordingly, the primary contribution of this study is to demonstrate the feasibility and operational potential of an adaptive, real-time visibility forecasting framework for a moving maritime platform, rather than to claim state-of-the-art performance against established maritime benchmarks. The results should therefore be interpreted as a proof-of-concept that highlights the benefits of continuous online adaptation, particularly under rapidly evolving and data-sparse marine environments.

Visibility in the training dataset follows the standard meteorological definition of horizontal visual range and is obtained from ICOADS as a daily baseline observation. For real-time training and evaluation, high-frequency visibility values are generated by combining this baseline with diurnal modulation linked to solar elevation, storm-proximity-based attenuation functions for storm scenarios, and controlled Gaussian noise to approximate sensor-level variability. In an operational deployment, these simulated values would be replaced directly by shipborne visibility sensors or drone-based optical measurements, without requiring any modification to the SeADL framework.

In addition, the current evaluation focuses on representative diurnal and storm-driven scenarios within a limited geographic region and is intended as a methodological demonstration rather than a comprehensive robustness assessment. While these cases are sufficient to illustrate the adaptive learning capability of the framework, broader validation across diverse maritime environments, storm events, and severities remains necessary. The system has not yet been deployed operationally, and validation using real shipborne and drone-based sensor measurements, particularly high-frequency observations, is an important direction for future work.

The forecasting task in this study is inherently short-horizon and adaptive. At each time step, the model predicts future visibility over a fixed horizon using only the information available at that moment. Model updates occur only after predictions are made and new observations become available, preserving a causal forecasting process. While this online adaptation allows the system to respond quickly to changing conditions, it can also introduce tracking-like behavior. Accordingly, the reported performance should be interpreted as predictive skill in a continuously adapting, real-time setting, rather than as that of a static model trained offline for long-horizon forecasting.

## 7. Conclusions and Future Work

In this study, we address the challenge of dynamic visibility forecasting in marine environments, where rapidly evolving conditions and limited data availability complicate accurate prediction. Visibility plays a critical role in maritime safety, operational efficiency, and navigation planning, indicating the need for forecasting approaches that can adapt in real time rather than relying solely on large, static historical datasets. To meet this need, we propose the SeADL framework, which performs continuous training and forecasting directly on streaming observations. Built on an LSTM-based architecture, the framework captures temporal dependencies through overlapping input sequences and supports real-time learning through minibatch training. This design enables the models to remain responsive to both short-term variability and longer-term environmental trends. The effectiveness of the proposed approach is demonstrated across multiple scenarios. The current-location model accurately captures diurnal visibility patterns, including sunrise and sunset transitions, while the remote-location models successfully forecast visibility under storm-influenced conditions. Although predictive performance degrades modestly when the vessel actively maneuvers around a storm, the models remain robust and informative throughout the navigation process. Overall, these results underscore the potential of real-time adaptive deep learning to support reliable visibility forecasting in complex and dynamic marine environments.

In future work, we plan to develop a fully dynamic, radar-style visualization to support simulated vessel navigation scenarios. This display will be updated in real time and color-coded based on visibility forecasts generated by the predictive models. In the current study, vessel trajectories were manually selected to ensure safe navigation; future extensions will incorporate AI-driven path-planning algorithms [41] that automatically identify safe routes using the most recent visibility predictions. Such capabilities will allow navigators to balance safety and operational efficiency according to mission requirements. We also aim to improve forecasting performance under dynamic navigation conditions by incorporating additional input features that better capture environmental variability. In addition, we plan to automate the selection of key model parameters, including sequence length and minibatch size, to reduce manual tuning and enhance overall model performance. At present, the learning rate remains fixed during real-time training; adaptive learning-rate schedules and regularization strategies will be explored to improve stability and accuracy over extended voyages [42,43]. Finally, we plan to investigate more advanced model architectures, including hybrid LSTM-CNN frameworks that integrate image-based inputs and Transformer-based models capable of capturing longer-range temporal dependencies [9]. These research directions are expected to further strengthen the robustness, scalability, and practical applicability of the proposed framework.

## Figures and Tables

**Table 1:** Selected input features for real-time marine visibility forecasting.

Input Feature	Unit	Source	Short Description
Precipitation	Millimeter	Onboard sensor/drone	Gridded rainfall measurement
Cloud Cover	Percentage	Onboard sensor/drone	Fraction of the grid covered by clouds
Dry-Bulb Temperature	Fahrenheit	Onboard sensor/drone	Gridded temperature measurement
Dew-Point Temperature	Fahrenheit	Onboard sensor/drone	Temperature at which air becomes saturated with water vapor
Surface Pressure	Inch of Mercury	Onboard sensor/drone	Proportional to the mass of air over the location
Wind Speed	Mile/hour (mph)	Onboard sensor/drone	Wind speed at current or remote locations
Wind Direction	Degree	Onboard sensor/drone	Wind direction at current or remote locations
Observed Visibility	Mile	Onboard sensors/drone	Current visibility used for future visibility forecasting
Solar Elevation	Degree	Derived from latitude, longitude, and time	Approximation of the Sun’s elevation (−90° to 90°)
Solar Elevation Cos/Sin	Unitless	Derived from solar elevation	Sine and cosine of solar elevation for smooth diurnal modeling

**Table 2:** Minibatch sizes and sequence lengths for diurnal and storm models.

Model Type	Sequence Length (*τ*)		Minibatch Size (*b*)	
	Tested Values	Chosen Value	Tested Values	Chosen Value
Diurnal Model	12, 24, 36, 72, 96	72	12, 36, 72, 144, 288, 576	576
Storm Model	3, 6, 12, 24, 36	6	1, 2, 3, 6, 9, 12	2

**Table 3:** Hyperparameter tuning for all SeADL models.

Hyperparameter	Tested Values	Chosen Value
LSTM Neurons	32, 64, 128, 256	256
Dense Neurons	32, 64, 128, 256	128
Dropout Rate	0%, 10%, 20%, 30%, 40%, 50%	30%
Optimizer	Adam, SGD, RMSProp	Adam
Activation Function	Leaky ReLu, Relu, Sigmoid	ReLu
Epochs (pre-training)	50, 100, 150, 200	100
Epochs (real-time training)	5, 10, 15, 20, 25, 30	10
Learning Rate (pre-training)	0.03, 0.01, 0.003, 0.001, 0.0003, 0.0001	0.0001
Learning Rate (real-time training)	0.0001, 0.00005, 0.00003, 0.00001	0.00005

**Table 4:** Monthly accuracy and MAE comparison for the current-location model.

Month	With Real-Time Training		Without Real-Time Training	
	Monthly Accuracy	Monthly MAE	Monthly Accuracy	Monthly MAE
March	94.91%	0.31 miles	84.97%	1.07 miles
April	95.02%	0.22 miles	78.87%	1.37 miles
May	96.45%	0.21 miles	79.39%	1.52 miles

**Table 5:** Sensitivity analysis on the noise parameters.

Noise Level	STD	MAPE	Accuracy
No Noise	0.0	6.70%	93.30%
Very Small Noise	0.05	6.83%	93.16%
Small Noise (*chosen value*)	0.2	7.82%	92.18%
Moderate Noise	0.5	8.62%	91.38%
High Noise	1.0	17.25%	82.75%
Very High Noise	2.0	32.61%	67.39%

## Data Availability

The data presented in this study are openly available from ICOADS at https://icoads.noaa.gov/ (accessed on 22 January 2025) and Copernicus Climate Data Store at https://cds.climate.copernicus.eu/ (accessed on 22 January 2025). The simulation data are not publicly available due to confidentiality agreements with the sponsor and restrictions related to joint research.

## Abbreviations

The following abbreviations are used in this manuscript:

ANN	Artificial Neural Network
API	Application Programming Interface
CNN	Convolutional Neural Network
DCNN	Deep Convolutional Neural Network
LSTM	Long Short-Term Memory
MAE	Mean Absolute Error
MAPE	Mean Absolute Percentage Error
MLP	Multilayer Perceptron
MSE	Mean Squared Error
NOAA	National Oceanic and Atmospheric Administration
RNN	Recurrent Neural Network
RT-SAC	Real-Time Self-Adaptive Classifier
SeADL	Self-Adaptive Deep Learning
